# Supplementary material for: Differences in Pulmonary Function Improvement after Once-Daily LABA/LAMA Fixed-Dose Combinations in Patients with COPD
Source: J Clin Med. 2022 Dec 1;11(23):7165. doi: 10.3390/jcm11237165 (PMC9739795; doi:10.3390/jcm11237165)
Supplement: Supplementary file 1 [file jcm-11-07165-s001.zip › jcm-2008613-supplementary.pdf]

Table S1. The baseline pulmonary function parameters for performing paired *t*-test.

	UMEC/VIL	GLY/IND	TIO/OLO	*p*-value
**FEV1, L (SD)**	1.38 (0.44)	1.41 (0.47)	1.41 (0.49)	0.868
**FEV1, % (SD)**	55.1 (16.4)	58.2 (15.7)	54.1 (16.8)	0.514
**FVC, L (SD)**	2.30 (0.57)	2.42 (0.58)	2.45 (0.66)	0.329
**FVC, % (SD)**	75.1 (16.7)	73.9 (16.3)	71.7 (16.4)	0.587
**RV, % (SD)**	173.8 (58.8)	176.8 (69.6)	180.1 (74.9)	0.898

CAT: COPD Assessment Test; CHF: Congestive heart failure; FEV1: Forced expiratory volume in 1 second; FVC: Forced vital capacity; GLY/IND: Glycopyrronium/Indacaterol; RV: Residual volume; SD: Standard deviation; TIO/OLO: Tiotropium/Olodaterol; UMEC/VIL: Umeclidinium/Vilanterol; FEV1, L UMEC/VIL $n = 108$, GLY/IND $n = 34$, TIO/OLO $n = 47$; FEV1, % UMEC/VIL $n = 111$, GLY/IND $n = 34$, TIO/OLO $n = 47$; FVC, L UMEC/VIL $n = 108$, GLY/IND $n = 33$, TIO/OLO $n = 47$; FVC, % UMEC/VIL $n = 67$, GLY/IND $n = 28$, TIO/OLO $n = 44$; RV, % UMEC/VIL $n = 81$, GLY/IND $n = 27$, TIO/OLO $n = 31$.
